# Clinical Utility of an Observation and Response Chart With Human Factors Design Characteristics and a Track and Trigger System: Study Protocol for a Two-Phase Multisite Multiple-Methods Design

**DOI:** 10.2196/resprot.3300

**Published:** 2014-08-12

**Authors:** Doug Elliott, Sharon McKinley, Lin Perry, Christine Duffield, Rick Iedema, Robyn Gallagher, Margaret Fry, Michael Roche, Emily Allen

**Affiliations:** ^1^Faculty of HealthUniversity of Technology SydneySydneyAustralia; ^2^Intensive Care UnitRoyal North Shore HospitalSydneyAustralia; ^3^South East Sydney Local Health District, and Faculty of HealthUniversity of Technology SydneySydneyAustralia; ^4^Agency for Clinical InnovationNSW HealthSydneyAustralia; ^5^Charles Perkins Centre and Sydney Nursing SchoolUniversity of SydneySydneyAustralia; ^6^Northern Sydney Local Health District, and Faculty of HealthUniversity of Technology SydneySydneyAustralia

**Keywords:** patient deterioration, track and trigger, rapid response system, observation charts, human factors design

## Abstract

**Background:**

Clinical deterioration of adult patients in acute medical-surgical wards continues to occur, despite a range of systems and processes designed to minimize this risk. In Australia, a standardized template for adult observation charts using human factors design principles and decision-support characteristics was developed to improve the detection of and response to abnormal vital signs.

**Objective:**

To describe the study protocol for the clinical testing of these observation and response charts (ORCs).

**Methods:**

We propose a two-phase multisite multiple-methods design to test the initial clinical utility of the charts in 10 hospitals of differing types and sizes across state jurisdictions in Australia. Data collection in the first phase includes user surveys, observations and field notes by project officers, handover de-briefs (short interviews with small groups of staff), and an audit of ORC documentation completion compared to the site’s existing observation chart. For the second phase, data will be collected using a retrospective audit of observation documentation from the previous hospital observation chart, prospective audit of observation documentation following implementation of the selected ORC, user focus groups, observational field notes, and patient outcome data from routinely collected organizational data sources.

**Results:**

Site selection and preparation, project officer training, chart selection and implementation, participant recruitment, and data collection has been completed and the analysis of these results are in progress.

**Conclusions:**

This detailed description of these study methods and data collection approaches will enable a comprehensive assessment of the clinical utility of these newly developed track and trigger charts and will be useful for clinicians and researchers when planning and implementing similar studies. Potential methodological limitations are also noted.

## Introduction

Widespread deficiencies in detecting and responding to clinical deterioration in adult patients in general wards at acute care hospitals continue despite a range of practice initiatives [1]. One key strategy to reduce serious adverse events has been the evolution from “cardiac arrest” teams to medical emergency teams (METs): in-hospital mortality rates now approximate 80% for cardiac arrests, 25% for MET calls, and 15% for patients with abnormal vital signs [2]. However, practices around the “afferent limb” of the rapid response system (RRS) (ie, patient monitoring, risk assessment, and event detection) remain identified areas for improvement [1]. At the core of these practices are patient observation charts.

Paper-based observation charts remain the dominant approach for documenting clinical observations of adult patients in acute general wards of Australian hospitals. With the continued identified failure to recognize and respond to signs of clinical deterioration evident [3], development and evaluation of charts have become a focus of recent work in Australia [4,5] and internationally [6-9].

The Australian Commission on Safety and Quality in Health Care (ACSQHC) implemented a program of work on “Recognizing and Responding to Clinical Deterioration” [10], focusing on ensuring that adult acute care medical-surgical patients whose clinical condition deteriorates receive appropriate and timely care and treatment. One initiative was to develop an evidence-based adult general observation chart that incorporated a system for recording patient observations, supporting accurate and timely recognition of clinical deterioration, and specifying prompt actions when deterioration was observed. Using human factors principles [11], the chart was designed to record physiological parameters (Element 1.6 of the National Consensus Statement; Respiratory Rate, Oxygen saturation, Heart Rate, Blood Pressure, Temperature, Consciousness Level), display thresholds for each physiological parameter or combination of parameters that indicate abnormality, specify the physiological parameters and other factors that trigger escalation of care, and include actions required when care is escalated [12].

The resulting adult deterioration detection system (ADDS) charts were designed with a multiparameter early warning scoring system (EWSS) using heuristic analysis [11] and were tested in a simulated environment [11,13-15]. The EWSS assigned scores from 0-5 for specified clinical parameters (described below), according to the level of derangement from normal, and then summed to produce a total score. A second version of the ADDS chart also included a separate table on the chart for scoring systolic blood pressure [16].

Three additional observation and response charts (ORCs) were subsequently developed by the ACSQHC after simulation testing to account for different track and trigger systems (TTS) across the full range of health services in Australia [12]. Each version used multiparameter vital signs alerts for clinical deterioration, and with one (Emergency Call), two (+ Clinical Review), or four levels (+ Senior Nurse Review, Increased Clinical Surveillance) of available clinical response. These additional charts were not tested in a simulated environment prior to the proposed clinical testing reported here.

**Figure 1:**
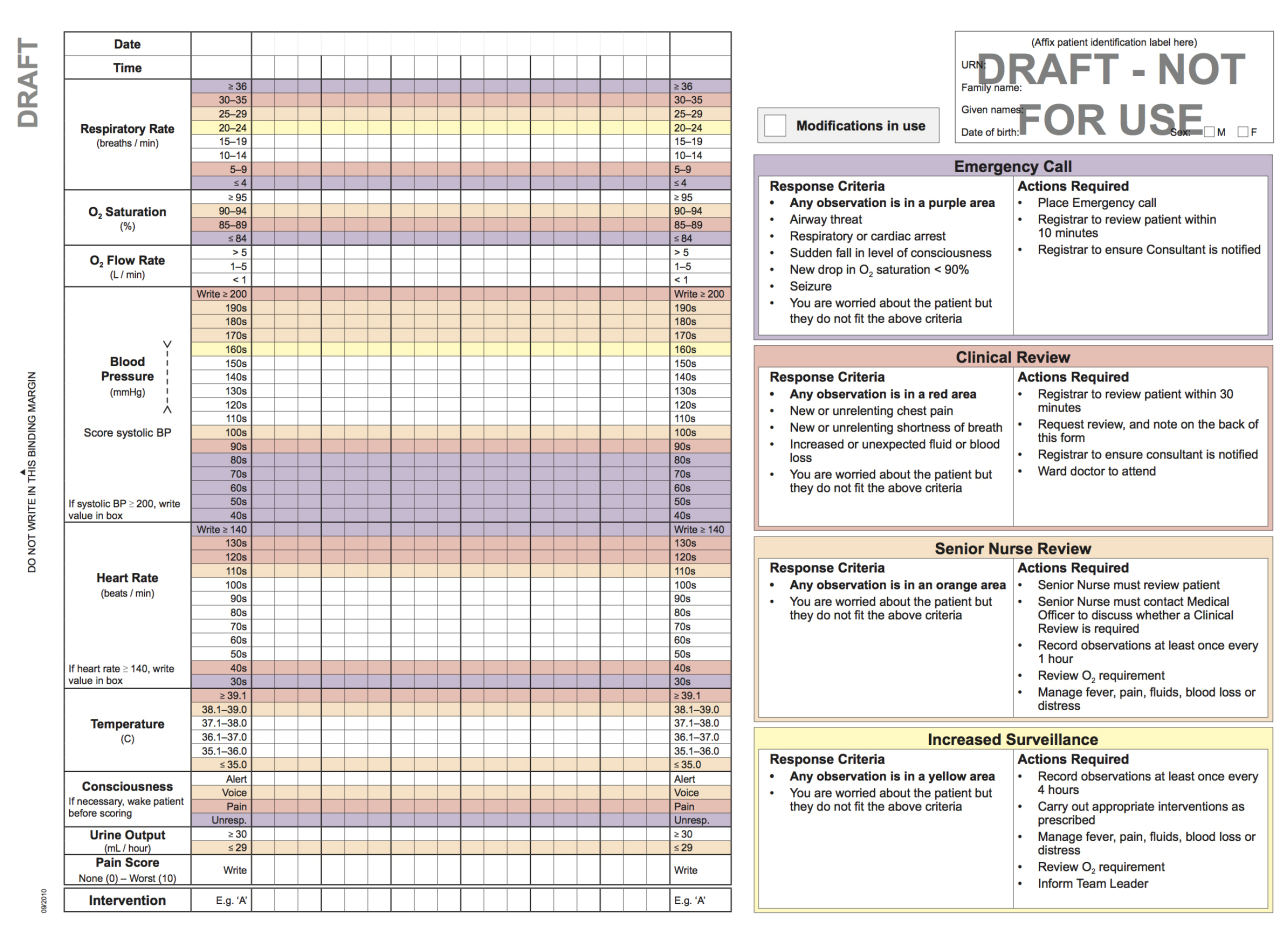
Example ORC, for 4-response level RRS.

Each version of the ORC is structured as a double-sided A3-sized form with a layout of a left binding margin and an off-center fold from the right. When folded, the cover page highlights to the user any other observation charts in use and modifications to parameter values for this patient. When folded out to the right, the inside left page contains the charting area for documentation of observations for nine specified parameters (in order from the top of the form: respiratory rate, oxygen saturation, oxygen flow rate, blood pressure, heart [pulse] rate, temperature, consciousness, hourly/4-hourly urine output, and pain score). All chart versions use the same graphing section [12]. Importance, not frequency, guided the location of each section in the chart. This charting area provided 18 columns for documenting observations. Every third column had a bold line to reduce “column-shift” error [17]. Each of the parameters had a range of normal values (with no shading) and a series of abnormal ranges with different colored shading, depending on the number of escalation response levels at each site. See Figure 1 for an example of a four-response level chart [12].

When graphing observations, users are instructed to place a dot (•) in the center of the box, which included the current observation in its range of values, and connect it to the previous dot with a straight line. For blood pressure, the “∨” and “∧” symbols are used for systolic and diastolic values respectively, and connected by a vertical dotted line. Pain score is the only parameter to use written numeric values, from 0 (none) to 10 (worst). When an observation is recorded in a shaded area, recommended actions are noted on the chart to guide clinicians to initiate an appropriate response, unless a modification has been documented previously on the ORC [12]. Use of colored bands was developed to delineate vital sign abnormalities, initiate a change in clinician behavior, and increase RRS triggering reliability using site-specific predefined actions [1].

The inside right page provides information only for the user, including the response criteria and actions required (it is not for writing information on). The final page contains sections to record interventions associated with abnormal vital signs, clinical review requests, and additional observations [12].

The aims and scope of our project are outlined in a competitive tender process from the ACSQHC. The first study aim is to test initial usability (ease of use) and clinical utility of the ORCs in general adult medical/surgical wards. The related objectives for this first phase are to examine whether the ORCs (1) are suitable for observations of adult medical-surgical patients and prompt a response for episodes of clinical deterioration, (2) have any sections that require modifications, and (3) could be introduced and applied in practice with minimal training.

Five versions of the charts [12] are available for testing in the clinical sites: (1) ADDS+: using an EWSS with a table for scoring systolic blood pressure, and four TTS (response) levels (Increased Clinical Surveillance, Senior Nurse Review, Clinical Review, Emergency Call), (2) ADDS-: using an EWSS without scoring systolic blood pressure, and four response levels (as above), (3) ORC R4: using a multiparameter TTS with four response levels (as above), (4) ORC R2: using a multiparameter TTS with two response levels (Clinical Review, Emergency Call), and (5) ORC R1: using a multiparameter TTS with one response level (Emergency Call).

The scope for this phase from the ACSQHC is for the charts to be used in parallel with existing hospital observation charts and tested in a small number of hospital sites of different types and size.

After modifications of the ORC templates (based on objective 2 above), the final study aim will be to examine whether the ORC templates demonstrated clinical utility when implemented into everyday clinical practice. The related study objectives are to investigate the (1) rate of completion of the ORC, (2) rate of abnormality in clinical observations, (3) rate of calling for assistance where indicated, and the response obtained, (4) preferences and comments of clinical staff, and (5) patient outcomes. The scope for this phase is to conduct a contained roll-out with site-customized ORCs implemented across a whole hospital/health service. As only one version is to be selected, implemented, and evaluated at each clinical site, no comparison of versions of the chart is planned. This paper describes the study protocol.

## Methods

### Design

**Figure 2:**
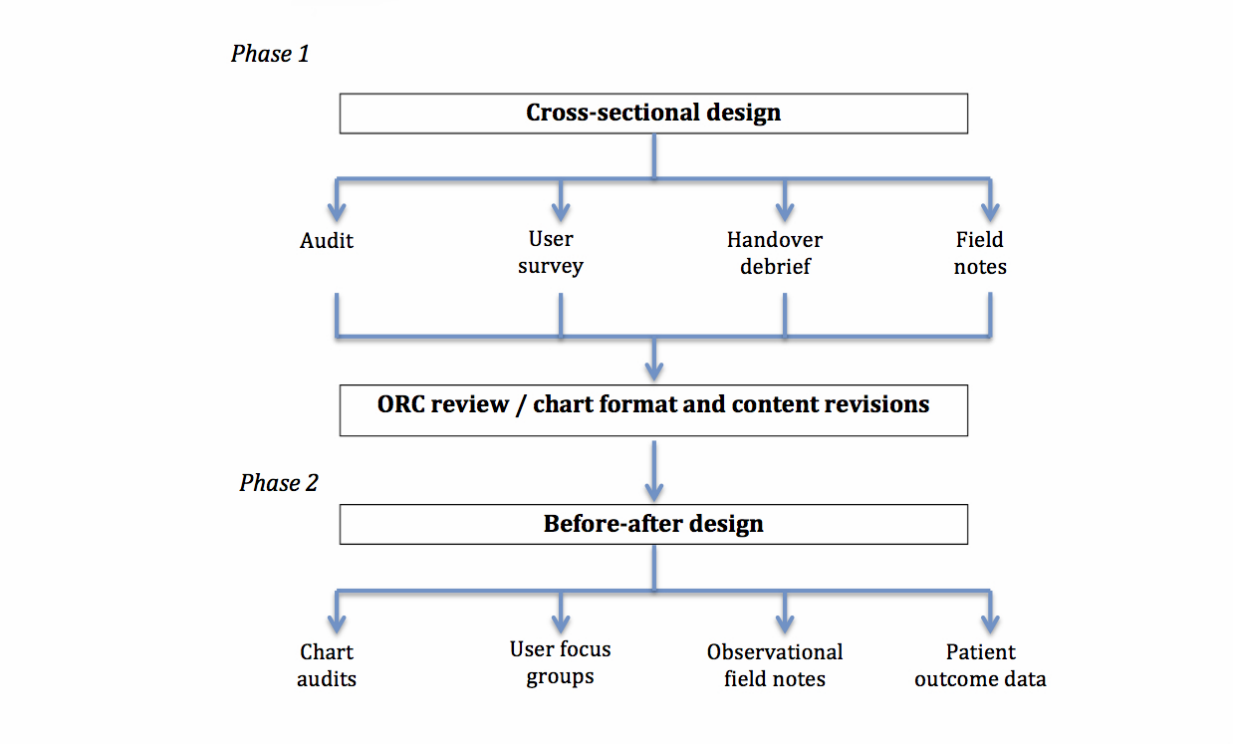
Study design.

A two-phase multiple-methods design was developed to examine usability of the selected ORCs in a range of adult clinical areas (see Figure 2). In Phase 1, initial clinical utility of the charts will be examined during a short implementation period, incorporating user surveys, observations, and field notes by project officers, handover debriefs (short interviews with small groups of staff), and an audit of ORC documentation completion compared to the site’s existing observation chart.

In the second phase, a before-after multiple-methods design will be used, with the selected chart version permanently implemented as the site observation chart. Proposed data collection includes retrospective audit of vital sign documentation from the hospital’s previous observation chart, prospective audit of vital sign documentation following implementation of the selected ORC, user focus groups, observational field notes, and patient outcome data from routinely collected organizational data sources.

### Site Selection and Sample

Expressions of interest will be sought from hospitals across all state jurisdictions using the ACSQHC contacts list. In the response, interested sites will confirm their ability to support implementation and evaluation of the ORC in their facility. Ten clinical sites will be selected in consultation with the ACSQHC. A sample will be selected that reflects differences in hospital size and level of service, preferred chart template, and location.

An executive from selected sites will be invited to engage as a champion for the project, liaising with key stakeholders, supporting the site-based project officer, and profiling the work with all relevant clinical staff. Site-based project officers will be seconded for the project and supported by training workshops, project manager site visits, teleconferences, telephone, and email assistance.

### Selection of Observation and Response Chart Version and Chart Modifications

Each site will select one of the five versions of the ORC that best matches their local escalation protocol and existing RRS for managing deteriorating patients. Each selected ORC template will then be modified to align parameter values with each site’s rapid response team calling criteria and RRS protocol and practices.

### Clinical Site Preparation

A site information package will be developed and distributed to each of the site executives and project officers and will also form part of the ethics application for each site (discussed below). The document will provide details of the different stages in this phase, as well as guidelines and tools for data collection, and different resources for the site-based project officer. A training workshop is planned to be conducted centrally for all project officers. A full-day event will provide project orientation, including the context of patient deterioration and the ACSQHC’s program of work, exploration of the ORC designs based on human factors development, introduction to the ORC Project and project team, and description of the data collection approaches using short demonstration videos, patient scenarios, and practice sessions.

Although training on completion of the chart for clinical staff is to be minimal as per the intent of the ACSQHC, staff preparation for data collection will be essential, and so each project officer will inform all relevant clinical staff (primarily nursing staff) about the new chart and the project. This is planned to include orientation to the design characteristics and components of the chart and the aims of the project and related data collection processes, specifically the need for dual-documentation of observations during the 24-hour data collection period. Given the issue of shift work and access to staff, this information will be provided in both written (information posters, information sheets in the communications folder or equivalent, email) and verbal forms (shift handovers preceding the data collection period, depending on staff roster patterns and practices).

For the required double documentation of patient observations, clinical staff will be requested to document on the hospital’s current observation chart first as per usual practice, as this forms part of a patient’s medico-legal record. They will then document the observations on the trial ORC during the same documentation activity, to minimize any variations between the two charts. On the designated data collection day for that ward, the project officer will distribute the selected chart for commencement at the start of the observation day (planned to be early afternoon).

### Data Collection Approaches

#### Phase 1

##### Summary

The multiple-methods approach for this initial phase will comprise an audit of the ORC for completeness of documentation of observations, compared to the hospital’s existing observation chart, a self-report survey by users, handover debriefs (short interviews with small groups of clinical staff), and observations and field notes from the site-based project officer.

Any addition to workload of clinical staff is a risk to study compliance and feasibility, and therefore data collection is planned to minimize respondent burden by scheduling each ward to complete the dual-documenting of observations on the existing hospital chart and the designated ORC only within one 24-hour period. A continuous 24-hour cycle of observations in each ward is most appropriate for testing the clinical utility of the charts, including assessment on the use of charts at night, when ambient lighting is lower. A staged process will be developed for each hospital site, so that data collection for each ward can be undertaken in sequential 24-hour periods, separated by a data collation day to allow completion of data collection from the previous ward and preparation for the next ward.

##### Audits

Dual documentation will be a requirement during this phase as the ORC will not be an approved medical record at this stage, and the current hospital chart will therefore remain in practice as part of the legal medical record during the trial. Following completion of the 24-hour period of dual-documentation data collection for each ward, each project officer will audit the ORCs for completeness of documentation of observations, compared to the hospital’s existing observation chart. These data will be entered via SurveyMonkey with guidelines provided to support the project officer. Compliance between the dual sets of documented observations will also be audited, comparing sets of vital signs on the ORC with sets of vital signs on the existing hospital chart to identify when (time of day) and where (variable on ORC) errors may occur. Any vital sign sets on the ORC that do not match the vital sign sets on the existing hospital chart will be considered as mismatched. Details of mismatched vital sign sets will be collected for a maximum of five sets per chart.

##### User Survey

A user-satisfaction survey will be completed by clinical staff at the end of their observation activities for the shift. The survey comprises 28 items relating to the design and components of the charts [15]. For ADDS charts, seven additional items relating to scoring and the blood pressure table will be included. Items will examine usability of the ORCs in the clinical setting, including clarity of text (size, font type), layout (size of chart, flow, and format of observation parameters), comprehensiveness, ease of documenting, and capacity to trigger a response for a deteriorating patient. Items are formatted as dichotomous and Likert-scale response levels for ease of completion. Demographic characteristics of each user will also be collected, including designation and qualifications of staff, employment type, and employment experience. Staff designation, particularly in relation to nursing or other care staff, is important to collect, given that the intent of the ORC is for it to be used by all levels of clinical staff undertaking patient observations without specific training.

Both paper-based and online versions of the survey will be developed, with each taking approximately 5 minutes to complete. Each project officer will distribute the paper-based surveys to users at the beginning of their shift and then collect the surveys at the time of user completion, to ensure an optimal return rate and completeness of the survey. For staff that prefer and have access to Internet-enabled computers in their work area, a site-unique link to SurveyMonkey will be provided. Only one user survey per participant will be completed.

##### Handover Debrief

At the completion of each shift (particularly after night duty), the project officer will conduct short interviews with a group of staff. These debrief sessions will be audio-taped with participant permission, for project officers to group or theme staff views and experiences. The aim of these interviews is to identify and explore the broad issues for clinical staff related to documentation in the ORC. Guidelines will be provided to support the project officer.

##### Field Notes

Each site-based project officer will document field notes while observing practices relating to the use of the selected chart. During peak periods of observation (eg, 1000, 1400, 1800 hours), the project officer will observe staff observation practices and communicate briefly with users for any anecdotal comments on the clinical utility of the charts.

##### Chart Modifications Following Phase 1

Following Phase 1 analyses and discussion with the chart developers, applicable modifications to the ORCs will be implemented prior to Phase 2. Any information and training issues for Phase 2 will be addressed by a range of information resources such as a project plan, posters, materials for use during in-service sessions, and a Frequently Asked Questions sheet, and supporting site-based project officers during the preparation and the roll-out of the ORCs into their settings.

#### Phase 2

##### Summary

The multiple methods to address this phase’s study objectives are a retrospective audit of current hospital observations charts and prospective audit of data following implementation of the selected ORC, user focus groups, observational field notes, and patient outcome data from routinely collected organizational data sources. After education and implementation of the ORCs in each site, clinical staff will use the charts routinely for observations for a minimum of 3 weeks, prior to data collection.

##### Retrospective and Prospective Audits

For the two audits, a 72-hour admission period was selected: February in the previous year (retrospective) and February in the current year (prospective). For each participating ward, 60 admission episodes will be audited at each site. Sunday, Monday, and Tuesday were selected as the audit period to include data related to activity occurring out of hours.

For the retrospective audit, observation charts in use prior to implementation of the ORC will be examined for rate of completion (number of complete sets of vital signs/number of charts audited), rate of recognition of abnormal clinical parameters (number of responses to an abnormal vital sign/number of abnormal vital signs identified on audit), and rate of triggered responses to a clinical deterioration (number of response teams triggered/number of clinical deteriorations identified on audit). Abnormal clinical parameters will be identified retrospectively using trigger criteria from the site-selected ORC. Data collection will also include hospital length of stay, location of discharge or transfer at end of admission, resuscitation status, and admission outcome.

During the prospective audit, the recently implemented ORCs will be audited for the same data as the retrospective audits, as well as extra items that will enable comparison with Phase 1 data on completion compliance according to the chart’s general instructions.

##### Focus Groups

After ORC implementation and a period of routine use, project officers will conduct short semistructured focus group interviews with clinical staff. Participant consent will be gained prior to data collection, and the focus groups will be audio-recorded for transcription of de-identified verbatim comments. Focus groups will be scheduled during shift overlap, staff development sessions, and education forums with the aim of capturing the views of as many staff comments as possible. Sample questions will be provided to each site project officer.

##### Observations of Documentation Practice

Field observations are planned for the site-based project officers at negotiated times with each clinical area piloting the ORC, for a recommended minimum of six observation sessions per selected ward over at least 1-2 hours duration during the prospective data collection period. Observation sessions will range across different shifts on different days, to enable observation of activities related to use of the ORC in routine observation practices. Guidelines and a template for field notes will support project officers’ observation of practices. Ward staff will be informed that observations related to ORC usage will occur using normal communication processes and visible placement of ward posters. Individual staff members are able to refuse participation during the observation periods, by negotiation with their ward manager. Project officer interaction with clinical staff is permissible during the observation period to clarify or ask a question.

##### Patient Outcome Data

To minimize data collection burden, patient outcome data will be collected using routinely collected organization-wide data systems for adverse events such as MET/arrest calls, unplanned intensive care unit admissions, unexpected deaths, and length of stay. These data will be collected for the months of the retrospective and prospective audit periods, as well as an annual summary for the previous year when available from sites.

##### Data Management and Analyses

In Phase 1, the project officer at each site will assess all quantitative data for completeness, before data entry either locally or centrally (for de-identified paper-based user surveys). All data will then be cleaned and checked for errors centrally by the project manager prior to data analysis. Qualitative interview and field notes data will be transcribed for analysis at each site and transmitted to the research team for collation prior to analyses.

Quantitative data from the user survey and audit will be analyzed descriptively using frequencies and proportions, for each site individually and for the total sample. Transcribed qualitative data from the field notes of observations, debrief sessions, and open-ended questions from the user survey will be entered into NVivo and examined initially via content analysis (where appropriate including counts of categories of text) and then thematic analysis. Coding of text will use categories aligned with the project aims; for example, clarity of text, chart format and layout, comprehensiveness, ease of documenting, and capacity to trigger a response for a deteriorating patient.

For Phase 2, all site data will be sent to the research team for management and analyses. Audit data will be entered into Microsoft Excel, then cleaned and coded for analysis in SPSS (version 19). Patient outcome data will be sent in original form from the sites and then re-formatted and coded in Microsoft Excel. For quantitative data, frequencies will be examined for distribution. Descriptive statistics will be used to examine all data. For non-normal distributions of continuous data, medians and interquartile ranges will be used. Categorical data will be presented using proportions and frequencies.

Focus groups will be audio-recorded, and sound files sent to the research team for transcription. Project officer field notes will be typed up as Microsoft Word documents and also sent to the research team. Qualitative data will be entered into NVivo 9 and analyzed for descriptive content and emerging themes.

##### Ethical Considerations

For Phase 1, we plan to submit the proposal to each clinical site as a negligible/low-risk project, given that clinical staff (not patients) are study participants and the level of risk entailed during data collection. Informed consent will be sought from participants (all relevant clinical staff) for the survey, observations, and interviews, as required, prior to data collection.

For Phase 2, a proposal will be initially submitted to the Human Research Ethics Committee (HREC) of one selected lead site. Once approval is given, applications will be submitted to the HRECs of all other participating sites as required by the relevant jurisdiction for each site. The university HREC will then be approached for ratification. Clinical staff participants will be asked to provide informed consent for the focus groups, and observation periods by the project officer, using the provided participant information sheet and informed consent form. Confidentially of participant identity will be assured. All data will be stored as per national regulatory guidelines [18].

## Results

Site selection and preparation, project officer training, chart selection and implementation, participant recruitment, and data collection has been completed and the analysis of these results are in progress.

## Discussion

### Study Summary

Clinical deterioration by adult patients in acute medical-surgical wards continues to occur, despite a range of systems and processes designed to minimize this risk. In Australia, the ACSQHC opted to develop a standardized template for adult observation charts using human factors design principles and decision-support characteristics to improve the detection of and response to abnormal vital signs.

This study aims to use a cross-sectional and a before-after design with multiple-methods data collection approaches to evaluate the implementation, clinical utility, and user acceptance of an observation and response chart for use with adult general medical-surgical patients in clinical sites across Australia. This pragmatic methodological approach aims to balance collection of a diverse dataset with a manageable level of participant burden, within the scope of the project tender set by the ACSQHC.

### Limitations

A number of potential limitations with these proposed methods are evident. The use of an onsite project officer seconded from the local organization’s staff will enable optimal communication and engagement with all relevant clinical staff. However, their involvement as data collectors, including facilitation of focus groups, has the potential to influence participant responses or behaviors (possible Hawthorne effect). The 24-hour cycle of data collection in Phase 1 is designed to enable involvement and feedback from night-duty staff. While data collection periods will be short, these are proposed to minimize participant burden in busy clinical environments.

The before-after design in Phase 2 may limit causal inferences related to the chart implementation. While the use of control wards in sites may have improved interpretation, this latter design would also have other potential limitations, including differing ward cultures, case mix, and contamination bias.

Within the context of the chart design characteristics, modifications to parameter values and response levels will enable alignment with local site needs, policies, and practices. This process of flexible standardization provides site input, but perhaps not from front line staff (the users). As noted earlier, while the three non-ADDS charts did not have any simulation testing prior to this clinical testing, the design characteristics and sections, including the graphing section, were similar across all versions [12]. Different versions of the charts will not be directly compared to each other in sites; while this was not a study aim, it will limit ability to identify any user preference for a particular chart version.

### Conclusions

This study plans to involve 10 sites from different Australian jurisdictions, including both public and private hospitals, with different levels of service and size, ranging from small rural facilities, to metropolitan and tertiary-level hospitals. This detailed description of the study methods will enable a comprehensive assessment of the clinical utility of these newly designed observation and response charts and will be useful for clinicians and researchers when planning and implementing similar studies.

## Abbreviations

ACSQHC: Australian Commission on Safety and Quality in Health Care
ADDS: adult deterioration detection system
EWSS: early warning scoring system
HREC: Human Research Ethics Committee
MET: medical emergency team
ORC: observation and response chart
RRS: rapid response system
TTS: track and trigger system
